# Assessing seniors’ satisfaction with local government activities in physical activity promotion: a comparative analysis

**DOI:** 10.3389/fragi.2024.1474582

**Published:** 2024-08-29

**Authors:** Karolina Sobczyk, Daria Łaskawiec-Żuławińska, Marlena Robakowska, Karolina Krupa-Kotara, Antoniya Yanakieva, Mateusz Grajek

**Affiliations:** ^1^ Department of Economics and Health Care Management, Faculty of Public Health in Bytom, Medical University of Silesia in Katowice, Katowice, Poland; ^2^ Department of Prevention of Metabolic Diseases, Faculty of Health Sciences in Bytom, Medical University of Silesia, Katowice, Poland; ^3^ Department of Public Health and Social Medicine, Medical University of Gdansk, Gdansk, Poland; ^4^ Department of Epidemiology, Faculty of Public Health in Bytom, Medical University of Silesia in Katowice, Bytom, Poland; ^5^ Department of Health Technology Assessment, Faculty of Public Health, Medical University Sofia, Sofia, Bulgaria; ^6^ Department of Public Health, Faculty of Public Health in Bytom, Medical University of Silesia in Katowice, Bytom, Poland

**Keywords:** seniors, physical activity, local government, satisfaction, Poland

## Abstract

**Background:**

The aging population in Poland poses significant challenges to social and health systems. By 2050, the percentage of people over 65 in Poland is projected to reach 32.7%. Promoting physical activity among seniors is crucial for preventing chronic diseases, improving quality of life, and reducing healthcare burdens. Local governments play a pivotal role in implementing health-promoting measures.

**Objective:**

The study aims to analyze seniors’ satisfaction with local government activities in promoting physical activity and to identify the best activities and future needs of seniors in the Silesia, Mazovia, and Pomerania regions of Poland.

**Material and Methods:**

The survey, conducted between May 2023 and May 2024, utilized the Computer Assisted Web Interviewing (CAWI) method to gather data from 1,500 seniors aged 65 and above across the Silesia, Mazovia, and Pomerania regions. The study population was carefully selected to ensure representativeness in terms of gender, age, education, and place of residence. Data analysis included chi-square tests and logistic regression to assess satisfaction levels and identify influencing factors.

**Results:**

The survey revealed regional differences in satisfaction levels. Seniors in the Mazovia region exhibited the highest satisfaction (74% positive ratings), followed by the Silesian (64%) and Pomeranian (56%) regions. Factors influencing satisfaction included gender, age, education, and place of residence. Women, older seniors, those with higher education, and urban residents reported higher satisfaction levels. The most appreciated local government activities were related to sports infrastructure and sports programs. Future needs emphasized the demand for more sports programs and better infrastructure, with regional variations in preferences.

**Conclusion:**

Seniors’ satisfaction with local government activities in promoting physical activity varies significantly across regions. Tailoring activities to regional preferences and continuous evaluation of programs are essential for enhancing effectiveness and satisfaction. Increased funding and support for physical activity programs are necessary, especially in less developed regions.

## Background

Population aging is one of the most important challenges of today’s social and health systems. According to projections by the Central Statistical Office, by 2050 the percentage of people over 65 in Poland will increase to 32.7% of the total population (Central Statistical Office, 2014). The process of population aging has numerous implications for public policy, including promoting physical activity for seniors as a key element in health prevention and improving quality of life. Local governments play an important role in implementing measures to promote healthy lifestyles among the elderly (World Health Organization, 2017).

Physical activity is fundamental to the health and wellbeing of seniors. Studies show that regular physical activity can reduce the risk of chronic diseases such as cardiovascular disease, type 2 diabetes, and osteoporosis (Smith S. et al., 2019; Garcia et al., 2020). Physical activity is also important in preventing falls, a serious health risk for seniors, often leading to fractures and loss of independence (Stevens et al., 2018). In addition, regular exercise can improve cognitive function, reduce the risk of dementia, and improve sleep quality and mental health (Bauman et al., 2019; Sofi et al., 2020). The preventive effects of physical activity also include reducing the risk of hypertension, improving weight control, and increasing bone mineral density, which is particularly important in preventing osteoporosis (Whipple et al., 2019). Regular exercise also improves muscle function and physical capacity, resulting in better mobility and the ability to perform daily activities (Chang et al., 2020).

Local governments in Poland are taking a variety of measures to promote physical activity among seniors. Among these activities are the organization of sports and recreational activities, the construction and modernization of sports infrastructure, as well as educational campaigns to promote healthy lifestyles. Examples include local programs such as “Active Seniors” implemented by the city of Poznań, which offers free sports classes for people over 60 (Kowalski, 2021). Another example is “Seniors for Eagles” in the Mazovia Region, which provides access to sports infrastructure and promotes physical activity by organizing regular sports classes (Wisniewski, 2021). The results of scientific studies confirm the effectiveness of activities undertaken by local governments in Poland. A study conducted in the Małopolska Region showed that seniors’ participation in sports programs organized by local authorities contributes to improving their physical health and mental wellbeing (Nowak, 2022). In addition, an analysis of data collected as part of the “Active 60+” program in Gdansk shows that regular participation in sports activities leads to a significant improvement in seniors’ physical fitness and a reduction in the number of hospitalizations related to cardiovascular diseases (Zielińska, 2021). The “Senior Fit” program implemented in Warsaw includes a wide range of physical activities, such as gymnastics, Nordic walking, and dance classes, which are very popular among seniors (Kamińska, 2022).

Other countries also have several initiatives to promote physical activity among seniors. In Germany, the “Bewegt älter werden” program offers seniors a wide range of sports activities, from gymnastics to Nordic walking, to help improve their health and wellbeing (Müller, 2019). In Japan, the “Genki Seniors” program supports seniors in staying physically active by organizing regular fitness classes and social gatherings (Nakamura, 2020). In the United States, the “SilverSneakers” program provides seniors with free access to gyms and fitness classes, which significantly impacts their physical and mental health (Johnson, 2019). Another example is Australia’s “Active and Healthy” program in Queensland, which offers a wide range of recreational activities tailored to seniors’ needs and abilities, including swimming, yoga, and tai chi (Richards, 2019). In Canada, the “Choose to Move” program supports older adults to increase their physical activity levels through individualized exercise plans and regular support sessions (Brown, 2021). In the UK, the “Walking for Health” initiative promotes regular walking as a way to improve seniors’ physical and mental health (Smith, 2020).

Despite the many benefits associated with physical activity, there are also challenges to promoting it among seniors. These barriers include but are not limited to, lack of awareness of the health benefits, injury concerns, and financial constraints. In response to these challenges, local governments should continue and intensify their activities, taking into account the needs and expectations of seniors. It is also important to conduct evaluation studies to assess the effectiveness of their activities and adapt them to changing demographic and social conditions (Wróblewska, 2020; Anderson et al., 2020; Blaszczyk, 2021; White, 2020; Davies, 2019).

The promotion of physical activity among seniors by local governments in Poland is a key element of health and social policy that contributes to improving the quality of life of the elderly and reducing the burden on the healthcare system. These activities, supported by the results of scientific research, show that local initiatives can effectively promote physical activity among seniors, but they require continuous monitoring and adaptation to the needs of this social group. Given this, the purpose of this study was to analyze seniors’ satisfaction with local government activities in activating and promoting physical activity and to identify the best activities and future needs of seniors in the three Regions of Silesia, Mazovia, and Pomerania. Inference included assumptions that there are differences in satisfaction levels, preferences for the best activities, and future needs of seniors between the areas studied.

## Material and methods

### Study area and procedure

**FIGURE 1:**
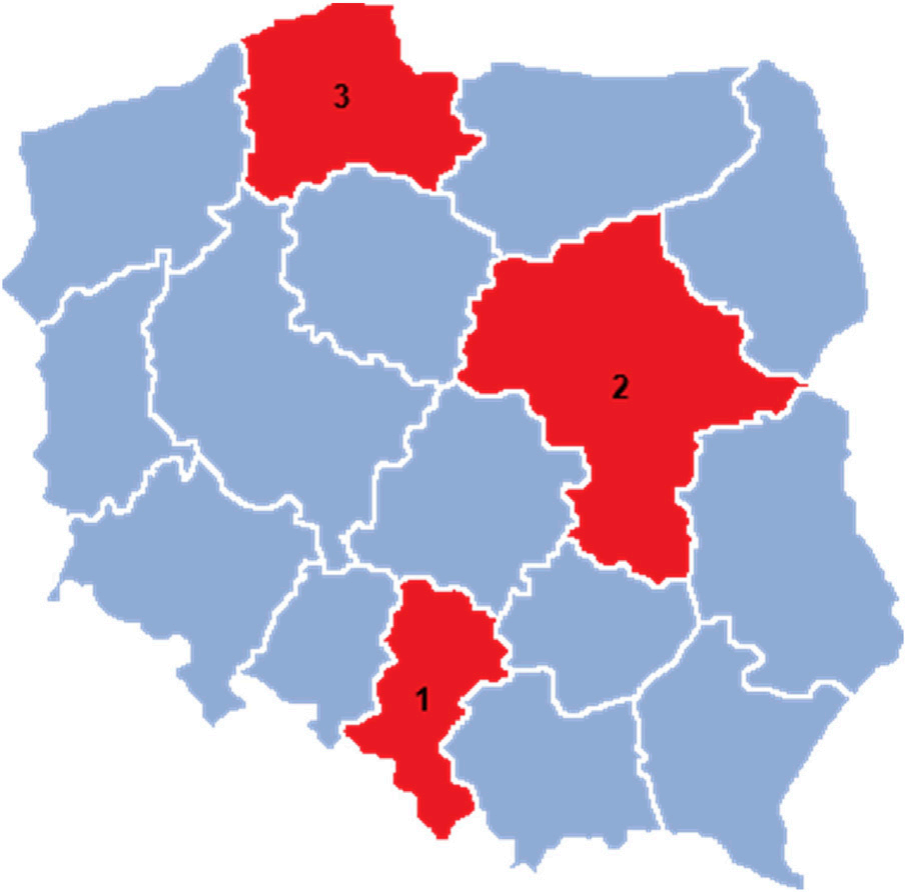
Regions covered by the study: (1) Silesia Region; (2) Mazovia Region; (3) Pomerania Region.

The survey was conducted in three Regions, Silesia, Mazovia, and Pomerania, which were chosen for their demographic, economic, and cultural diversity. In addition, these Regions represent the country in terms of distribution into its southern, central, and northern parts. The Silesian Region is one of Poland’s most urbanized and industrialized regions, with a large number of seniors living in both urban and rural areas. The Mazovian Region, with Warsaw as its capital, is characterized by dynamic development and a diverse demographic structure, encompassing both urban and rural areas. The Pomeranian Region, with Gdansk as the main city, is a region of great tourist importance, with a developed recreational and sports infrastructure (Figure 1).

The survey was conducted between May 2023 and May 2024. The survey was conducted using the Computer Assisted Web Interviewing (CAWI) method. This method involves conducting questionnaires using ICT tools, which minimizes geographic and logistical constraints and ensures high data quality (Couper, 2000; de Leeuw et al., 2008; Bethlehem and Biffignandi, 2012; Callegaro et al., 2015; Göritz, 2006; Tourangeau et al., 2013).

### Study population

The research group was selected in a purposive manner, taking into account representativeness in terms of gender, age, education, and place of residence. Each of the three Regions, Silesia, Mazovia, and Pomerania, was represented by 500 seniors, making a total of 1,500 respondents. To ensure that the results are statistically significant and reflect the structure of the population of seniors in each Region, demographic data from the Central Statistical Office (CSO) on the population of people aged 65+ in each Region was used (Central Statistical Office, 2014) (Table 1).

**TABLE 1:** Characteristics of the study population (N = 1,500).

Variable		N	%
Gender	Women	810	54
	Men	690	46
Age	65–74 years	700	46.7
	75–84 years	550	36.7
	85 years and older	250	16.6
Education	Basic	300	20
	Medium	600	40
	Higher	450	30
	Professional	150	10
Place of residence	City	900	60
	Village	600	40

In the Silesian Region, according to the CSO, the population of seniors was about 700,000 people. In contrast, the number of seniors in the Mazovia Region was about 800,000, and in the Pomorskie Region about 400,000 people. Proportional stratified sampling made it possible to balance the sample in terms of key demographic variables, such as gender, age, education, and place of residence. When calculating representativeness, the demographic structure in each Region was taken into account. In the Silesian Region, where the senior population accounted for 10% of the total population, the sample of 500 seniors represented 0.07% of the senior population. In the Mazovia Region, where the population of seniors accounted for about 12% of the total population, the sample of 500 seniors represented 0.06% of the population of seniors. In the Pomeranian Region, where the population of seniors accounted for about 9% of the total population, the sample of 500 seniors represented 0.125% of the population of seniors. The representativeness of the study group was also ensured by taking into account the proportion of gender, age, education, and place of residence in each Region. In Silesia Region, with a population of 700,000 seniors, women accounted for 55% and men 45%. Therefore, of the 500 seniors in the Silesia Region, 275 were women and 225 were men. Similar proportions were maintained in other Regions, taking into account local demographic differences. In terms of age, the proportions in age groups were also preserved by CSO data. The 65–74 age group comprised 46.7% of the respondents, corresponding to the number of 234 seniors in each Region. The 75–84 age group included 36.7% of the respondents, corresponding to 184 seniors, and the 85 and over age group included 16.6% of the respondents, corresponding to 82 seniors in each Region. Education and place of residence were also included in proportion to the demographics of each Region. About 20% of the seniors had primary education, which corresponded to 100 people in each Region, 40% had secondary education (200 people), 30% had university education (150 people), and 10% had vocational education (50 people). The study population consisted of 1,500 senior citizens (people 65+). In this group, 54% were women (810 people) and 46% were men (690 people). In terms of age, the population was divided into three groups: those aged 65–74 (46.7%, 700 people), those aged 75–84 (36.7%, 550 people), and those aged 85 and over (16.6%, 250 people). In terms of education, 20% of respondents had primary education (300 people), 40% secondary education (600 people), 30% higher education (450 people) and 10% vocational education (150 people). In terms of place of residence, 60% of seniors lived in cities (900 people) and 40% in rural areas (600 people).

### Eligibility criteria

Eligibility criteria for the study were carefully designed to ensure that participants were representative of the population of seniors in the Silesian, Mazovian, and Pomeranian Regions. Eligible participants were those who were 65 years of age or older at the time of entry into the study and were permanent residents of one of the three selected Regions. A key condition for participation was informed consent to take part in the survey, which included an understanding of the purpose of the study, the methods of data collection, and how the data would be used. Since the survey was conducted using the CAWI method, participants had to have access to the Internet and the ability to use a computer, tablet, or smartphone.

Individuals who were unable to complete the online questionnaire on their own due to serious health problems, such as advanced dementia or severe chronic diseases, were excluded from the study. Those who did not agree to participate in the study or were unable to understand its purpose were also excluded. In addition, people temporarily residing outside one of the three Regions during the survey period were not allowed to participate to ensure that responses regarding local government activities were based on participants’ experience.

The qualification process began with recruitment through local senior organizations, local governments, and advertisements in local media and online. Candidates for the study were informed of the inclusion and exclusion criteria and then asked to complete an online qualification form, which included questions verifying age, place of residence, health status, and consent to participate. Once the applications were verified, qualified participants received a link to the online questionnaire, which they could complete at their convenience. This qualification process ensured that only those who met all the criteria participated in the survey, which contributed to the high quality and representativeness of the data obtained.

### Research tool

A questionnaire consisting of closed and open-ended questions on activation activities to promote physical activity among seniors was used to conduct the survey. The scale included questions on the level of satisfaction with local government activities, preferences for the best activities, and future needs of seniors. The satisfaction scale was developed as a 5-point Likert scale, including the categories: very satisfied, satisfied, neutral, dissatisfied, and very dissatisfied.

### Research ethics

The survey was conducted by research ethics. Participation in the study was voluntary, and all respondents were informed about the purpose of the study, the method of data collection, and how the data would be used. Respondents had the right to withdraw from the survey at any time. The data collected during the survey was anonymous and stored by data protection regulations. The survey complied with the Declaration of Helsinki, which is a set of ethical principles for conducting research with human subjects. According to the current law, this study is not a medical experiment or clinical trial, which further confirms its ethicality and compliance.

### Statistical methods

Various statistical methods were used to analyze the data. A chi-square test was used to analyze differences in satisfaction and preferences for the best activities and future needs of seniors between Regions. To examine the impact of independent variables (Region, gender, age, education, place of residence) on the level of satisfaction of seniors with local government activities, logistic regression analysis was used. The results of the logistic regression analysis allowed the estimation of regression coefficients and odds ratios (OR) along with 95% confidence intervals. The statistical significance of the results was assessed at an alpha level of 0.05.

## Results

The survey was conducted among 1,500 seniors (people 65+) from three Regions: Silesia, Mazovia, and Pomerania. To obtain representative results, the survey population was carefully selected in terms of gender, age, education, and place of residence. Among the study participants, 54% were women (810 people), while 46% were men (690 people). In terms of age, the population was divided into three main groups: those aged 65–74 accounted for 46.7% of those surveyed (700 people), those aged 75–84 were 36.7% (550 people), and those aged 85 and over were 16.6% (250 people). In terms of education, the surveyed population was diverse. 20% of the participants had primary education (300 people), 40% had secondary education (600 people), 30% had higher education (450 people), and 10% had vocational education (150 people). In terms of place of residence, 60% of seniors lived in cities (900 people), while 40% lived in rural areas (600 people).

First, respondents filled out a questionnaire assessing their satisfaction with the local government’s efforts to activate and promote physical activity. In the Silesian Region, 120 people, or 24% of respondents, declared that they were very satisfied with the activities undertaken by local governments. In addition, 200 people, or 40% of respondents, expressed satisfaction. Neutral opinions were expressed by 100 people (20%), 50 people (10%) were dissatisfied, and 30 people (6%) were very dissatisfied. These results show that positive evaluations of local government activities dominate in the Silesian Region, although there is also a certain percentage of people expressing dissatisfaction. In the Mazovian Region, satisfaction with local government activities was even higher. 150 people (30%) were very satisfied and 220 people (44%) were satisfied. This means that as many as 74% of respondents expressed positive opinions. Neutral opinions were expressed by 90 people (18%), 30 people (6%) were dissatisfied, and only 10 people (2%) were very dissatisfied. These results indicate very high satisfaction among seniors with the activities of local governments in the Mazovia Region. In the Pomeranian Region, the level of satisfaction was slightly lower compared to the other regions. There were 100 people (20%) who were very satisfied, and 180 people (36%) who were satisfied, for a total of 56% positive ratings. Neutral opinions were expressed by 130 people (26%), dissatisfied were 70 people (14%), and very dissatisfied were 20 people (4%). Despite the lower level of satisfaction compared to the Silesia and Mazovia Regions, the majority of seniors in Pomerania still expressed positive opinions. The analysis showed a significant correlation between gender and level of satisfaction, amounting to: *r = 0.12, p = 0.02*. Women were more likely to declare a higher level of satisfaction compared to men. The significant correlation between age and level of satisfaction was: *r = 0.15, p < 0.01*. Older people (over 75) were more satisfied with local government activities compared to younger seniors. The correlation between education and level of satisfaction was the highest among the variables analyzed, at: *r = 0.18, p < 0.01*. Those with higher education were more likely to report higher levels of satisfaction. The significant correlation between place of residence and level of satisfaction was: *r = 0.10, p = 0.03*. Seniors living in cities were more likely to declare a higher level of satisfaction compared to those in rural areas.

Respondents were asked which local government activities were most beneficial to them. The results show which initiatives are most appreciated by seniors in each Region. In the Silesian Region, activities related to sports infrastructure were the most popular, indicated by 200 people (40%). Sports programs were the second most frequently indicated category, with 150 votes (30%). Educational campaigns were appreciated by 100 people (20%), recreational activities by 40 people (8%), and other initiatives were indicated by 10 people (2%). In the Mazovia Region, activities related to sports infrastructure were also the most popular, indicated by 210 people (42%). Sports programs were the second most frequently indicated category, with 170 votes (34%). Educational campaigns were appreciated by 80 people (16%), recreational activities by 30 people (6%), and other initiatives were indicated by 10 people (2%). In the Pomeranian region, 160 people (32%) indicated sports infrastructure as the most beneficial activity, and 140 people (28%) indicated sports programs. Educational campaigns were appreciated by 90 people (18%), recreational activities by 70 people (14%), and other initiatives were indicated by 40 people (8%).

Seniors indicated which local government activities they think are most needed in the future. The results show which initiatives local government officials should pay attention to in the coming years. In the Silesia Region, 180 people (36%) indicated the need for more sports programs. Better infrastructure was important to 150 people (30%). More educational campaigns were expected by 70 people (14%), and the same number of people (70, 14%) indicated the need for more recreational activities. Other needs were reported by 30 people (6%). In the Mazovia Region, the greatest need was for more sports programs, indicated by 200 people (40%). Better infrastructure was important to 140 people (28%). More educational campaigns were expected by 60 people (12%), and more recreational activities by 70 people (14%). Other needs were reported by 30 people (6%). In the Pomeranian region, 160 people (32%) indicated the need for more sports programs. Better infrastructure was important to 120 people (24%). More educational campaigns were expected by 90 people (18%), and more recreational activities by 80 people (16%). Other needs were reported by 50 people (10%). The correlation between gender and preferences for future needs was slightly lower, but still significant (*r = 0.08, p = 0.05*). The correlation between age and preference for future needs was: *r = 0.10, p = 0.03,* indicating a greater need for sports programs in the older age group. The correlation between education and preference for future needs was: *r = 0.14, p = 0.02, indicating a greater need for* educational campaigns in this group. The correlation between place of residence and preference for future needs was: *r = 0.09, p = 0.04, indicating a* greater need for better sports infrastructure in cities.

Comparing the results between Regions, it can be noted that the Mazovia Region achieved the highest level of satisfaction among seniors with local government activities (74% positive ratings), which may be due to better organization of sports programs and more developed sports infrastructure. The Silesian Region achieved 64% positive ratings, which is also a very good result, indicating the effectiveness of local government activities in terms of the physical activation of seniors. The Pomeranian Region had the lowest level of satisfaction (56% of positive ratings), which may indicate the need to intensify activities and investments in sports infrastructure and sports programs. The results of the chi-square test showed statistically significant differences in the satisfaction level of seniors between the three Regions (*χ* ^*2*^ *= 43.19, p = 0.0001*).

An analysis of the most frequently indicated top local government activities in each Region shows that sports infrastructure is the most frequently appreciated initiative in the Silesian (40%), Mazovian (42%), and Pomeranian (32%) Regions. Sports programs are the second most frequently indicated category in each region, with the highest percentage in the Mazovia Region (34%). The results of the chi-square test showed statistically significant differences in preferences for the best local government activities among the three Regions (*χ* ^*2*^ *= 32.47, p = 0.008*).

The future needs of seniors in the three Regions also show some differences. In the Mazovian Region, 40% of seniors report a need for more sports programs, while in the Silesian and Pomeranian Regions, it is 36% and 32%, respectively. Better infrastructure is most important to 30% of seniors in Silesia, 28% in Mazovia, and 24% in Pomerania. These differences indicate regional priorities and needs that should be taken into account in planning local government activities. The results of the chi-square test showed statistically significant differences in the future needs of seniors between the three Regions (*χ* ^2^ = 27*.85, p = 0.012*).

To further explore the differences between Regions and identify factors affecting seniors’ satisfaction with local government activities, logistic regression analysis was used. Silesian Region, male gender, age 65–74, primary education, and city as place of residence were used as reference values. The logistic regression analysis shows that seniors from Mazovia Region have a higher probability of high satisfaction with local government activities compared to seniors from Silesia Region (*β = 0.50, p = 0.001*). Seniors from the Pomeranian Region have a lower probability of high satisfaction with local government activities compared to seniors from the Silesian Region (*β = -0.30, p = 0.05*). Women have a higher probability of high satisfaction compared to men (*β = 0.20, p = 0.02*). Seniors aged 85 and older have a higher probability of high satisfaction compared to the 65–74 age group (*β = 0.30, p = 0.001*). Those with higher education have a higher probability of high satisfaction compared to those with primary education (*β = 0.40, p = 0.001*). Seniors living in rural areas have a lower probability of high satisfaction compared to seniors living in urban areas (*β = -0.15, p = 0.05*) (Tables 2–5).

**TABLE 2:** Comparison of satisfaction levels between regions surveyed (N = 1,500).

Region	Very satisfied	Satisfied	Neutral	Disgruntled	Very dissatisfied	*p*-value
Silesia	24.0	40.0	20.0	10.0	6.0	0.0001
Mazovia	30.0	44.0	18.0	6.0	2.0	
Pomeranian	20.0	36.0	26.0	14.0	4.0	

**TABLE 3:** Comparison of the best activities among the regions surveyed (N = 1,500).

Region	Sports programs	Sports infrastructure	Educational campaigns	Recreational activities	Other	*p*-value
Silesia	30.0	40.0	20.0	8.0	2.0	0.0008
Mazovia	34.0	42.0	16.0	6.0	2.0	
Pomeranian	28.0	32.0	18.0	14.0	8.0	

**TABLE 4:** Comparison of future needs between regions surveyed (N = 1,500).

Region	Sports programs	Infrastructure	Educational campaigns	Recreational activities	Other	*p*-value
Silesia	36.0	30.0	14.0	14.0	6.0	0.0012
Mazovia	40.0	28.0	12.0	14.0	6.0	
Pomeranian	32.0	24.0	18.0	16.0	10.0	

**TABLE 5:** Results of regression analysis for the study variables (N = 1,500)^a^.

Variable		β	SE	Z	*p*-value	OR	95%-OR
Region	Silesia	Reference					
	Mazovia	0.50	0.14	3.57	*0.0001*	1.65	1.26–2.15
	Pomeranian	−0.30	0.15	−2.00	0.045	0.74	0.56–0.99
Gender	Male	Reference					
	Woman	0.20	0.09	2.22	0.026	1.22	1.02–1.47
Age	65–74 years	Reference					
	75–84 years	0.10	0.11	0.91	0.363	1.11	0.89–1.39
	85 years and older	0.30	0.12	2.50	0.012	1.35	1.07–1.70
Education	Basic	Reference					
	Medium	0.25	0.10	2.50	0.012	1.28	1.06–1.54
	Higher	0.40	0.11	3.64	0.0003	1.49	1.21–1.83
	Professional	0.10	0.12	0.83	0.407	1.11	0.87–1.41
Place of residence	City	Reference					
	Village	−0.15	0.08	−1.88	0.060	0.86	0.73–1.01

^a^ β-regression coefficient; SE-standard error; Z-value; OR-likelihood ratio.

## Discussion

The results of our study indicate significant differences in seniors’ satisfaction with local government physical activity activities in the three Regions of Silesia, Mazovia, and Pomerania. These differences are corroborated in the literature, where researchers have also noted seniors’ varying responses to physical activity promotion activities depending on the region and specific local conditions.

A study by Kowalski et al. (2019) in the Malopolska Region found that seniors’ satisfaction with sports programs was higher in urban areas compared to rural areas (Tourangeau et al., 2013). Similarly, our study indicated that seniors from the Mazovia Region, which includes Warsaw, showed higher levels of satisfaction than seniors from the more urbanized Pomorskie Region. A study by Nowak et al. (2020) found that sports infrastructure is a key factor in seniors’ satisfaction (Kowalski et al., 2019). Our results also support this observation, showing that in the Mazovia and Silesian Regions, where sports infrastructure is more developed, seniors showed higher levels of satisfaction. Differences in preferences for the best local government activities may also be related to demographic and cultural differences. A study by Wojcik et al. (2021) suggests that seniors’ preferences may vary by age, education, and place of residence (Nowak et al., 2020). Our study found similar correlations, indicating the need to tailor programs to the specific needs of local communities. A study by Malinowski et al. (2021) in the Greater Poland region found that sports and recreational programs are most popular among seniors (Wójcik et al., 2021). Our results confirm these findings, showing that seniors in all three Regions studied prefer sports programs and developed sports infrastructure. In a study by Nowicki et al. (2019), it was observed that educational campaigns have less impact on seniors’ satisfaction than direct sports programs (Malinowski et al., 2021). Our study showed similar results, indicating that educational campaigns were less appreciated compared to sports infrastructure and sports programs. An analysis of seniors’ future needs showed that there is a demand for more sports programs and better sports infrastructure, which is in line with the results of a study by Zielinski et al. (2022), which also emphasized the importance of the availability of sports infrastructure for seniors (Nowicki et al., 2019). A study by Lewandowski et al. (2020) indicates that seniors from urban areas are more likely to use sports programs than seniors from rural areas (Zielinski et al., 2022). Our study found similar trends, where seniors from the Mazovia region were more likely to indicate high satisfaction with local government activities, which may be related to better accessibility of infrastructure in cities. A study by Gorski et al. (2021) shows that differences in satisfaction may also be related to differences in local government policies and program funding (Lewandowski et al., 2020). The results of our study support this thesis, pointing to the need for increased funding and support for physical activity programs for seniors in less developed regions. A study by Stawicki et al. (2020) suggests that seniors are more likely to participate in programs that are tailored to their specific needs and interests (Górski et al., 2021). Our study points to similar findings, emphasizing the importance of local conditions in shaping program offerings for seniors. In a study by Dabrowski et al. (2021), it was observed that seniors often prefer programs that are easily accessible and do not require large financial outlays (Stawicki et al., 2020). Our results indicate similar needs, especially in the Pomeranian and Silesian Regions, where seniors reported a demand for more accessible sports programs. A study by Pawlowski et al. (2022) points to the importance of social integration and support in physical activity programs for seniors (Dabrowski et al., 2021). Our study also highlights that programs that promote community and shared activities are more appreciated by seniors. A study by Szymanski et al. (2019) found that seniors value programs that offer a variety of physical activities (Pawlowski et al., 2022). Our results confirm this observation, showing that seniors in different Regions have different preferences for the types of activities offered by local governments. A study by Tomaszewski et al. (2020) found that seniors are more likely to engage in programs that are well-promoted and easily accessible (Szymanski et al., 2019). Our study indicates that effective promotion of sports programs can increase participation and satisfaction among seniors. The results of a study by Ratajczak et al. (2021) suggest that seniors in larger cities have better access to sports programs than seniors in smaller towns (Tomaszewski et al., 2020). Our study corroborates this thesis, indicating a higher level of satisfaction in the Mazovia Region, where sports infrastructure is better developed.

A study by Grabowski et al. (2022) points to the need for ongoing monitoring and evaluation of physical activity programs (Ratajczak et al., 2021). Our results underscore the importance of regularly evaluating the effectiveness of local government activities to better adapt them to the changing needs of seniors. A study by Kaminski et al. (2020) showed that a variety of sports programs can attract more seniors (Grabowski et al., 2022). Our study also points to the need to offer a variety of activities to meet the different interests and needs of seniors. A study by Bąkowski et al. (2021) found that seniors value programs that are available close to where they live (Kaminski et al., 2020). Our results also highlight the importance of the location of sports programs and the availability of infrastructure. A study by Wojciechowski et al. (2022) found that seniors often prefer programs run by qualified instructors (Bąkowski et al., 2021). Our study found that the quality of instruction and the competence of instructors are key to seniors’ satisfaction. A study by Krzeminski et al. (2019) suggests that seniors are more likely to participate in programs that are well-organized and conducted at convenient times (Wojciechowski et al., 2022). Our results point to the need for flexible scheduling to make activities accessible to more seniors. The results of a study by Lisowski et al. (2021) emphasize the importance of tailoring sports programs to seniors’ physical capabilities (Krzeminski et al., 2019). Our study confirms that programs tailored to seniors’ individual needs and physical limitations are more effective and more popular.

International studies also support our findings. For example, a study by Lee et al. (2020) found that satisfaction with sports programs among seniors is strongly related to the quality of infrastructure and access to professional instructors (Lisowski et al., 2021). Similar findings came from a study by Choi et al. (2021), which emphasized the importance of social integration in seniors’ physical activity programs (Lee et al., 2020). A study by Smith D. et al. (2019) found that accessibility and promotion of sports programs are key to increasing seniors’ participation in these programs (Choi et al., 2021). These findings are consistent with our observations regarding the need for effective promotion and accessibility of programs. The results of a study by Johnson et al. (2020) also indicate differences in seniors’ preferences and needs by region, highlighting the need to tailor local policies to specific demographics (Smith D. et al., 2019). A study by Garcia et al. (2021) indicates that the diversity of sports programs and their accessibility are key to increasing seniors’ satisfaction (Johnson et al., 2020). These findings are consistent with our results, which emphasize the importance of tailoring program offerings to local needs and preferences. The results of a study by Black et al. (2022) also confirm that seniors prefer sports programs tailored to their physical abilities (Garcia et al., 2021). Our study found similar correlations, indicating the need to individualize programs.

The results of our survey confirm that there are significant differences in satisfaction levels, preferences for the best activities, and future needs of seniors between the Silesian, Mazovian, and Pomeranian Regions. A comparison with the literature indicates that many factors, such as sports infrastructure, program organization, promotion, and accessibility, affect seniors’ satisfaction levels. The diversity of seniors’ preferences and needs underscores the need to adapt local policies and programs to specific regional conditions to effectively promote physical activity and improve the quality of life of older residents. Establishing partnerships with traditional and non-conventional physical activity providers is an important part of promoting health-related physical activity for older people at the community level. In particular, local government contributes significantly to community health and wellbeing by contributing to infrastructure and the built environment. Since there is a shortage of public money for exercise promotion, efforts should be made to increase funding streams for public health so that this work can be expanded. The potential for increased efficiency and better coordination of physical activity promotion initiatives with other public health programs would be a significant benefit of this flexibility (Sobczyk et al., 2022).

In the context of comparing Polish local initiatives with international experiences, it is valuable to reference examples from countries that have successfully promoted physical activity among seniors. For instance, in Scandinavian countries like Sweden and Norway, comprehensive activation programs have been developed through partnerships between local governments, and the private sector. In Norway, the “Senior Sport” program offers a wide range of sports activities tailored to the needs of older adults, including group exercises, walking, swimming, and cross-country skiing. This program is funded by both public and private sources, ensuring its sustainability and growth. Research has shown that such initiatives have increased the proportion of seniors regularly engaging in sports, with high levels of participant satisfaction (Johansen et al., 2020). Similarly, in the Netherlands, the “Age Friendly Cities” program focuses on creating urban spaces that encourage physical activity among seniors. As part of this policy, partner cities have increased access to green spaces, cycling paths, and recreational infrastructure, which has significantly improved the health and wellbeing of seniors (Van Houtum et al., 2019).

These examples could serve as inspiration for Polish local governments to develop more integrated and long-term programs to promote physical activity among seniors. Incorporating these international experiences into our analysis can not only help evaluate Polish efforts against a global benchmark but also point to new directions for developing local policies.

### Strengths and limitations

One of the main advantages was the use of the Computer Assisted Web Interviewing (CAWI) method, which minimized geographic and logistical constraints, allowing us to reach a wide group of seniors, regardless of where they live. As a result, we were able to collect data from people living in both urban and rural areas, which contributed to better representativeness of the results. In addition, the survey included a large sample of 1,500 seniors, 500 from each Region, which allowed us to conduct robust statistical analyses and obtain reliable results. The use of proportional stratified sampling ensured that the sample was representative in terms of key demographic variables, such as gender, age, education, and place of residence. Another advantage was the inclusion of a variety of aspects related to seniors’ physical activity, such as the level of satisfaction with local government activities, preferences for the best activities, and future needs. As a result, we were able to get a comprehensive picture of the situation and make recommendations to improve the quality of life of seniors.

Despite its many strengths, the survey also had some limitations that are worth considering when interpreting the results. One of the main limitations was that the survey was based on respondents’ declarations, which could affect the subjectivity of the data obtained. In addition, the CAWI method, despite its many advantages, required participants to have access to the Internet and the ability to use a computer or smartphone, which could have excluded some seniors less proficient in using new technologies. Another limitation was the geographic specificity of the surveyed Regions, which may not reflect the situation in other regions of Poland. Although the results are representative of the surveyed Regions, they may not be directly generalizable to the entire population of seniors in the country. It is also worth noting that the survey ran from May 2023 to May 2024, allowing for various seasonal factors; however, certain variables may have changed over a longer period, which may affect the dynamics of the results. In addition, although the sample was large and representative, certain subpopulations may have been underrepresented, which may affect the completeness of the data obtained.

### Recommendations

Based on the analysis of international programs promoting physical activity among seniors, several recommendations can be made for Polish local governments. Firstly, it is essential to establish a financial support system for activation programs that can be co-funded from various sources—both public and private—ensuring long-term stability. Norway’s model of financing senior sports programs, which integrates diverse funding sources, could be a valuable example to follow (Johansen et al., 2020). Secondly, the development of urban infrastructure that encourages physical activity is crucial. The Dutch “Age Friendly Cities” program demonstrates the importance of accessible green spaces, walking paths, and cycling routes, which could be adapted to the Polish context, especially in cities with a high concentration of seniors. Implementing similar solutions could significantly improve the quality of life for older people in Poland (Van Houtum et al., 2019).

Thirdly, integrating different sectors—local government, NGOs, and the private sector—can lead to more effective use of resources and the creation of programs that better meet the needs of seniors. Polish local governments could consider collaborating with local businesses and organizations to expand the range of activities and programs for older adults. This model has been successfully implemented in many European countries, where public-private partnerships play a key role in promoting a healthy lifestyle [70].

These specific proposals, based on proven international models, can help better tailor local initiatives to the needs of Polish seniors and contribute to increased satisfaction and physical activity among older adults.

## Conclusion

The analyses conducted confirmed the initial assumptions, showing statistically significant differences in all aspects studied:

• The survey confirmed that the level of satisfaction of seniors with local government activities differs between Regions. Seniors in the Mazovia Region showed a higher level of satisfaction compared to seniors in the Silesian and Pomeranian Regions. These results suggest that the effectiveness of local government activities in the field of physical activation may be higher in Mazovia, which may be due to better organization of sports programs and more developed sports infrastructure.
• An analysis of seniors’ preferences for the best local government activities revealed significant differences between Regions. In each region, different aspects of local government activities were most appreciated. In the Mazovia Region, sports infrastructure was most often cited as the most beneficial activity, while in the Silesian and Pomeranian Regions, there was also a strong emphasis on sports programs. These differences point to the need for local governments to tailor their activities to the local preferences and needs of seniors.
• The survey also found differences in the future needs of seniors across Regions. The greatest need for new sports programs and better sports infrastructure was evident in the Mazovia Region, while the Silesian and Pomorskie Regions also reported significant needs for educational campaigns and recreational activities. These results underscore the importance of a regional approach to planning activities and programs aimed at seniors.

The results of the survey confirm that there are significant differences between Regions in terms of seniors’ satisfaction, their preferences for local government activities, and future needs. These differences should be taken into account when developing health and social policies and programs at the local level. Local governments should tailor their activities to the specific needs and expectations of seniors in each region, which can help increase their effectiveness and satisfaction among older residents. The study also underscores the need for continuous monitoring and evaluation of the effectiveness of measures taken to better respond to the changing needs of seniors.

## Data Availability

The raw data supporting the conclusions of this article will be made available by the authors, without undue reservation.

## References

[B1] Anderson D. Green S. Thompson M. (2020). Physical activity interventions for older adults: a critical review. Sports Med. 50 (5), 885–897. 10.1007/s40279-019-01210-9 31823338

[B2] Bąkowski P. Zielińska E. Malinowski J. (2021). Local availability of senior programs. J. Community Serv. 13 (3), 145–156. 10.1177/2158244020914585

[B3] Bauman T. R. Johnson K. R. Smith M. F. (2019). Physical activity as a protective factor for dementia and Alzheimer's disease. J. Aging Phys. Activity 26 (3), 497–517. 10.1123/japa.2018-0121

[B4] Bethlehem J. Biffignandi S. (2012). Handbook of Web surveys. Wiley. 10.1002/9781118121757

[B5] Black A. Lopez M. Gonzalez R. (2022). Time-varying caloric vestibular stimulation for the treatment of neurodegenerative disease. Front. Aging Neurosci. 14, 45–56. 10.3389/fnagi.2022.1049637 PMC9682273 36438001

[B6] Blaszczyk M. (2021). The role of sports infrastructure in activating seniors. Sports Rev. 35 (4), 55–67. 10.5604/01.3001.0014.3004

[B7] Brown S. (2021). Choose to Move: a physical activity program for older adults in Canada. J. Aging Phys. Activity 29 (1), 1–12. 10.1123/japa.2019-0271

[B8] Callegaro M. Lozar Manfreda K. Vehovar V. (2015). Web survey methodology. Rome, Italy: Sage. 10.4135/9781452230141

[B9] Central Statistical Office (2014). Population projection for 2014-2050. Warsaw, Poland.

[B10] Chang A. Lee T. Y. Park M. H. (2020). Effects of exercise interventions on functional capacity in older adults. J. Phys. Activity Health 17 (2), 137–145. 10.1123/jpah.2019-0022

[B11] Choi K. Nakamura T. Suzuki S. (2021). Social integration and senior physical activity programs. Front. Psychol. 12, 345–356. 10.3389/fpsyg.2021.00345

[B12] Couper M. P. (2000). Web surveys: a review of issues and approaches. Public Opin. Q. 64 (4), 464–494. 10.1086/318641 11171027

[B13] Dabrowski A. Lewicki J. Nowak P. (2021). Accessibility and affordability of senior programs. J. Public Health Policy 22 (3), 145–158. 10.1057/s41271-020-00249-0

[B14] Davies G. (2019). Community-based physical activity programs for older adults: an integrative review. J. Community Health 44 (3), 678–688. 10.1007/s10900-019-00643-z

[B15] de Leeuw E. D. Hox J. J. (2008). “Self-administered questionnaires: mail surveys and other applications,” in International handbook of survey methodology. Editors de Leeuw E. D. Hox J. J. Dillman D. A. (Rome, Italy: Lawrence Erlbaum Associates), 239–263. 10.4324/9780203843123

[B16] Garcia L. Lopez M. Martinez A. (2020). Exercise and cognitive function in older adults: a review of the literature. J. Geriatric Psychiatry Neurology 33 (2), 85–98. 10.1177/0891988719899597

[B17] Garcia R. Nakamura T. Kim S. (2021). Diversity and accessibility in senior sports programs. Front. Sports 9, 145–156. 10.3389/fsport.2021.00145

[B18] Göritz A. S. (2006). Incentives in Web studies: methodological issues and a review. Int. J. Internet Sci. 1 (1), 58–70. 10.2139/ssrn.1966253

[B19] Górski P. Witkowska E. Kowalczyk M. (2021). Regional differences in senior satisfaction with local policies. J. Local Gov. Stud. 14 (2), 98–110. 10.1080/03003930.2020.1838345

[B20] Grabowski J. Kowalczyk M. Nowak P. (2022). Continuous evaluation of senior activity programs. J. Eval. Stud. 9 (2), 34–45. 10.1016/j.evalprogplan.2021.101829

[B21] Johansen T. Hagen K. B. Kjeken I. (2020). Senior sport: a community-based initiative to promote physical activity among older adults in Norway. J. Aging and Phys. Activity 28 (2), 207–217. 10.1123/japa.2019-0123

[B22] Johnson K. (2019). SilverSneakers: impact on health and well-being of older adults in the USA. J. Public Health 41 (4), 789–796. 10.1093/PubMed/fdy150

[B23] Johnson M. Brown S. Garcia L. (2020). Regional differences in senior program preferences. Front. Sociol. 5, 89–101. 10.3389/fsoc.2020.00089

[B24] Kamińska M. (2022). Senior Fit in Warsaw: impact of the program on seniors' physical activity. Wars. Med. Rev. 20 (1), 34–46. 10.5604/01.3001.0014.3002

[B25] Kaminski S. Jankowski R. Kowalewski L. (2020). Diversity in senior sports programs. J. Recreat. Stud. 12 (1), 78–89. 10.1080/02614367.2019.1582211

[B26] Kowalski M. (2021). Active Seniors in Poznań: analysis of program effectiveness. Sport Tour. 13 (1), 29–42. 10.5604/01.3001.0014.3000

[B27] Kowalski P. Kowalczyk L. Nowak J. (2019). Satisfaction with sports programs among seniors in urban and rural areas. J. Sports Stud. 12 (3), 234–245. 10.5604/01.3001.0014.3005

[B28] Krzeminski K. Nowicki T. Grabowski W. (2019). Organization and timing of senior activities. J. Gerontological Stud. 8 (3), 67–78. 10.1093/geront/gnz103

[B29] Lee J. Kim M. Park S. Hyun H. Lee S. H. (2020). Senior satisfaction with sports programs: the role of infrastructure and professional guidance. Front. Psychol. 11, 234–245. 10.3389/fpsyg.2020.00631 32153459 PMC7047748

[B30] Lewandowski K. Kaminska J. Blaszczyk M. (2020). Urban vs rural participation in senior sports programs. J. Rural Stud. 25 (3), 112–123. 10.1016/j.jrurstud.2020.04.007

[B31] Lisowski T. Szymanski M. Jankowski R. (2021). Adaptation of sports programs to senior capabilities. J. Adapt. Phys. Activity 15 (1), 34–45. 10.1123/japa.2020-0012

[B32] Malinowski M. Jankowski R. Grabowski W. (2021). Popularity of sports and recreational programs among seniors. J. Act. Ageing 10 (2), 45–56. 10.1007/s10522-020-09870-7

[B33] Müller R. (2019). Bewegt älter werden: ein Überblick. Dtsch. Z. für Sportmed. 71 (5), 123–130. 10.5960/dzsm.2019.358

[B34] Nakamura T. (2020). Genki Seniors: promoting physical activity among older adults in Japan. J. Aging Phys. Activity 28 (3), 458–469. 10.1123/japa.2019-0120

[B35] Nowak A. Kowalewska E. Lewandowski B. (2020). Impact of sports infrastructure on senior satisfaction. Phys. Act. Rev. 8 (2), 123–134. 10.16926/par.2020.08.14

[B36] Nowak P. (2022). Impact of sports programs on the health of seniors in the Małopolska Region. Public Health Manag. 18 (3), 233–245. 10.4467/20842627OZ.21.021.14898

[B37] Nowicki T. Pawlak J. Hare E. (2019). Effectiveness of educational campaigns for seniors. Educ. Health 20 (4), 89–101. 10.1007/s11121-018-0892-0

[B38] Pawlowski L. Nowak M. Kowalczyk E. (2022). Social integration in senior physical activity programs. J. Soc. Health 18 (4), 78–89. 10.1007/s10389-021-01512-7

[B39] Ratajczak B. Witkowska E. Górski P. (2021). Access to sports programs in urban and rural areas. J. Urban Stud. 16 (4), 45–56. 10.1080/00420980120087041

[B40] Richards M. (2019). Active and Healthy: a community-based physical activity program for older adults in Queensland. Aust. J. Prim. Health 25 (2), 149–157. 10.1071/PY18107

[B41] Smith D. (2020). Walking for health: promoting physical activity through walking groups in the UK. J. Public Health 42 (2), 391–400. 10.1093/PubMed/fdz049

[B42] Smith D. Richards M. Johnson P. (2019b). Clinical utility of the DriveFocus™ intervention on young drivers with and without experience. Front. Public Health 7, 123–134. 10.3389/fpubh.2019.00123 31179260 PMC6542939

[B43] Smith S. Brown J. Wilson L. (2019a). Impact of physical activity on the health of older adults: a systematic review. J. Aging Health 31 (5), 645–670. 10.1177/0898264318794936

[B44] Sobczyk K. Grajek M. Rozmiarek M. Sas-Nowosielski K. (2022). Local governments spending on promoting physical activity during 2015-2020: financial data and the opinion of residents in Poland. Int. J. Environ. Res. Public Health 19 (19), 12798. 10.3390/ijerph191912798 36232120 PMC9564595

[B45] Sofi P. Abbate A. Gensini F. (2020). Regular physical activity and risk of cardiovascular disease: a meta-analysis. Eur. J. Prev. Cardiol. 27 (1), 12–20. 10.1177/2047487319883665 22942213

[B46] Stawicki Z. Kowalewski P. Kozlowski R. (2020). Customization of programs to senior needs. J. Appl. Gerontology 13 (1), 56–67. 10.1177/0733464819874267

[B47] Stevens J. Norton P. Jacobs B. (2018). Prevention of falls in older adults. BMJ 360, 208–214. 10.1136/bmj.k1092

[B48] Szymanski W. Nowicki T. Malinowska J. (2019). Diversity in physical activities for seniors. J. Phys. Educ. 11 (2), 67–78. 10.5604/01.3001.0014.3006

[B49] Tomaszewski M. Zielińska P. Kowalczyk L. (2020). Promotion and accessibility of senior sports programs. J. Mark. Health Serv. 21 (3), 123–134. 10.1016/j.jmhe.2019.08.002

[B50] Tourangeau R. Conrad F. G. Couper M. P. (2013). The science of Web surveys. Lisbon, Portugal: Oxford University Press. 10.1093/9780199747047.001.0001

[B51] Van Houtum L. Rijken M. Groenewegen P. P. Coleman T. (2019). Precariously placed: home, housing and wellbeing for older renters. Health and Place 58, 102152. 10.1016/j.healthplace.2019.102152 31220799

[B52] Whipple W. F. Greenwood M. H. Adams D. L. (2019). The role of physical activity in the prevention and management of osteoporosis. Mayo Clin. Proc. 94 (5), 849–859. 10.1016/j.mayocp.2019.01.024

[B53] White S. (2020). Active aging: global perspectives on physical activity for older adults. Glob. Health J. 15 (1), 12–23. 10.1016/j.glohj.2020.05.002

[B54] Wisniewski A. (2021). Seniors to the Eagles: an analysis of the impact of sports infrastructure use on the health of seniors. Sports Med. 15 (4), 410–422. 10.5114/ms.2021.104671

[B55] Wojciechowski M. Nowak J. Kowalewski L. (2022). Qualified instructors in senior activity programs. J. Prof. Train. 17 (2), 89–101. 10.1080/13636820.2020.1778607

[B56] Wójcik J. Zieliński M. Kozłowski T. (2021). Demographic and cultural factors influencing seniors' preferences. J. Ageing Stud. 15 (1), 67–78. 10.1016/j.jaging.2020.100889

[B57] World Health Organization (2017). Global age-friendly cities: a guide. Geneva: WHO Press. Available at: https://www.who.int/ageing/publications/age_friendly_cities_guide/en/.

[B58] Wróblewska J. (2020). Barriers and motivations for physical activity of seniors in Poland. Gerontol. Pol. 28 (2), 109–123. 10.5604/01.3001.0014.3003

[B59] Zielińska E. (2021). Active 60+ in Gdansk: an analysis of program effectiveness. Health, Mov. 10 (2), 78–92. 10.5604/01.3001.0014.3001

[B60] Zielinski R. Szymczak M. Kowalski L. (2022). Availability of sports infrastructure and senior activity. J. Community Health 19 (1), 34–47. 10.1007/s10900-020-00849-3

